# Impact of the Teaching Nursing Home on Nursing Student’s Willingness to Work in Long-Term Care

**DOI:** 10.1093/geroni/igaf122.3961

**Published:** 2025-12-31

**Authors:** Howard Degenholtz, Nancy Zionts, Anny Treat, Elizabeth Schlenk, Erin Kitt-Lewis, Lori Ingleton Gold, Kenna Campbell, Chelsea Dickson

**Affiliations:** University of Pittsburgh, Pittsburgh, Pennsylvania, United States; Jewish Healthcare Foundation, Pittsburgh, Pennsylvania, United States; University of Pittsburgh, School of Public Health, Pittsburgh, Pennsylvania, United States; University of Pittsburgh, Pittsburgh, Pennsylvania, United States; Penn State, State College, Pennsylvania, United States; University of Pennsylvania, Philadelphia, Pennsylvania, United States; University of Pittsburgh, School of Public Health, Pittsburgh, Pennsylvania, United States; Jewish Healthcare Foundation, Pittsburgh, Pennsylvania, United States

## Abstract

The Teaching Nursing Home Collaborative (TNHC) aims to strengthen the long-term care workforce and improve quality of care. The initiative integrates several key components: clinical rotations for nursing students in partnered nursing homes, implementation of the Institute for Healthcare Improvement’s Age-Friendly Health System “4Ms” framework (What Matters, Mobility, Mentation, Medication), and an online learning collaborative. This study reports on the experience of nursing students in select courses from three schools of nursing who participated in clinical rotations at four nursing homes from 2022 to 2024. Students reported their interest in working with older adults and in long-term care settings and rated their competencies at the beginning and end of the semester: interdisciplinary collaboration, conducting resident assessments, eliciting resident values, health promotion, resident interviewing, medication review, assessing what matters most, and conducting an evidence-based project. School of nursing faculty instructors evaluated student competencies at the end of the term. Analysis included 438 student responses at baseline and 521 at the end of the semester, along with 290 faculty assessments. Student self-reported competence significantly improved across all eight domains (p < .001). Overall, faculty rated students significantly lower than the students’ self- ratings. Over time, faculty ratings of student competence increased (p < .001). Students reported relatively weak preferences for working with older adults or in long-term care post-graduation. This preference did not change over time. These findings suggest that while the TNH program is meeting pedagogical goals, additional strategies are needed to influence student attitudes toward careers in long-term care.

